# Effects of Lung Support Formula on respiratory symptoms among older adults: results of a three-month follow-up study in Shanghai, China

**DOI:** 10.1186/1475-2891-12-57

**Published:** 2013-05-06

**Authors:** Yong Cai, Rong Shi, Huijiang Song, Meili Shang, Tian Shen, Mina Shariff, Kenneth Kami, Pingping Gu, Tuong Nguyen, Jianyu Rao

**Affiliations:** 1 School of Public Health affiliated with Shanghai Jiaotong University School of Medicine, Shanghai 200025, People’s Republic of China; 2 Sanlin Community Sanitary Service Center, Pudong New Area, Shanghai 200025, People’s Republic of China; 3 Department of Research, DRM Resources, 1683 Sunflower Avenue, Costa Mesa, CA 92626, USA; 4 Department of Pathology and Laboratory Medicine, David Geffen School of Medicine, University of California at Los Angeles, Los Angeles, CA 90095, USA

## Abstract

**Background:**

With the acceleration of industrialization in low or middle-income nations, the prevalence of respiratory symptoms among older adults is even more significant now in China. Contemporary treatments using Western medicine, such as anti-inflammatory regimens, may be effective in relieving the symptoms, but may have unexpected side effects. Some natural products may be effective in improving respiratory functions, yet their efficacies remain to be examined in randomized, placebo-controlled studies. To evaluate the effects of Lung Support Formula, a nutritional supplement which contains naturally derived Chinese herbal medicines, we conducted a clinical study among older adults in Shanghai, China.

**Methods:**

A total of 100 patients over 50 years old were recruited and blindly randomized into the treatment or control group. The subjects took either 1 Lung Support Formula capsule or a placebo capsule twice a day for 12 weeks. All subjects were followed-up every 4 weeks to perform investigative and clinical examinations. Repeated measure of analysis of variance was employed to compare the trend of respiratory symptoms scores between the 2 groups during 12 weeks of follow-up.

**Results:**

Fifty patients from the treatment group and 49 patients in the control group completed the 3-month follow-up. No adverse events were reported in the treatment duration. The percentage of patients reported to have chronic cough, chronic expectoration and chronic bronchitis were significantly decreased in the treatment group when compared with baseline after a 3-month intervention (*P* < 0.05). The respiratory symptoms scores declined gradually with the lapse of time (*P* < 0.05) in the treatment group and there were no significant changes in the control group by repeated measure of analysis of variance (*P* > 0.05).

**Conclusions:**

The clinical research shows that use of Lung Support Formula shows significant improvements of respiratory symptoms and is well-tolerated in short-term use among older adults. An additional study involving more subjects and longer-term follow-up would be needed to provide convincing evidence of the improvement of respiratory symptoms in the treatment group.

## Background

Smoking and air pollution are major causes of respiratory problems such as chronic bronchitis and other respiratory syndromes [1,2]. Indoor and outdoor air pollution such as cooking smoke [3], traffic and industrial pollutants are some of the most serious environmental and public health problems in the industrialized world [4,5], which contributes to the prevalence of respiratory symptoms including chronic pharyngitis, laryngitis, tracheitis, bronchitis and chronic obstructive pulmonary disease (COPD) [6,7]. With the acceleration of industrialization in low or middle-income nations, the problem is even more significant now in China. Preliminary evidence was provided in some research that elderly people might be more vulnerable to air pollution than younger adults [8,9]. Contemporary treatments with Western medicine, such as anti-inflammatory regimens, etc., may be effective in relieving symptoms associated with chronic bronchitis, but may have unexpected side effects [10-12]. Some natural products may be effective in improving respiratory functions, yet their efficacies remain to be examined in randomized, placebo-controlled studies [13-15].

Lung Support Formula is a nutritional supplement which contains naturally derived *Astragalus membranaceus*, *Cordyceps sinensis*, *Ophiopogon japonicus*, *Panax ginseng*, *Morus alba*, *Ginkgo biloba*, *Prunus armeniaca*, *Forsythia suspensa*, *Salvia miltiorrhiza* and *Gekko gecko*, plus vitamin A, vitamin C, magnesium and zinc. The main ingredients of Lung Support Formula are traditional herbs used for invigoration, health preservation and reduction of fatigue in Traditional Chinese Medicine for thousands of years [16]. Astragalus was used to strengthen the body and cordyceps was verified that it could improve exercise performance and might contribute to wellness in older subjects [15]. Ophiopogon was used to treat certain respiratory conditions by stimulating production of mucus, making coughs more productive. However, the benefits of this supplement in relieving respiratory symptoms have not been reported in a clinical setting. To evaluate the effects of Lung Support Formula on improving respiratory symptoms in older adults, we conducted a clinical study in Shanghai, China.

## Methods

### Study participants

Study subjects were recruited from the Health Service Center of Sanlin community in Shanghai, China. The study was reviewed and approved by the Internal Review Boards of Sanlin Community Health Service Center, and all participants gave written, informed consent before enrollment. The criteria for including people in the study were as follows: 1) over 50 years old, either male or female; 2) had symptoms like chronic cough, expectoration, dyspnea for more than 1 month; 3) had chronic pharyngitis, laryngitis, tracheitis, bronchitis or COPD. Enrolled patients must have met the first criteria and any of the other two criteria mentioned above. Subjects were excluded if they had any history of: 1) cancer; 2) other serious diseases such as cardiovascular disease, etc.; 3) acute inflammatory symptoms or flu, etc.; 4) had used any prescription or OTC drugs to treat respiratory disease recently (within the past 30 days).

### Design and sample size

The present study was a double-blind, placebo-controlled clinical trial. Subjects were provided with Lung Support Formula or identical placebo capsules. The Lung Support Formula capsules were manufactured following current Good Manufacturing Practices (cGMP) guidelines by Robinson Pharma, Inc. (Orange County, CA, USA). The main ingredients of the Lung Support Formula capsules included *Astragalus membranaceus*, *Cordyceps sinensis*, *Ophiopogon japonicus*, *Panax ginseng*, *Morus alba*, *Ginkgo biloba*, *Prunus armeniaca*, *Forsythia suspensa*, *Salvia miltiorrhiza*, *Gekko gecko*, vitamin A, vitamin C, magnesium and zinc. The placebo was also manufactured by Robinson Pharma with the main ingredients being Calcium, Maltodextrin, and Rice Flour.

The sample size was calculated according to our hypothesis that the incidence of chronic cough (*P*_1_) in the control group is 90%. The incidence in the intervention group (*P*_2_) decreased to 70%. We set α level as 0.05, β level as 20%. The pretest probability (1-β) was 90%. The formula used to calculate sample size is shown below:

$$\bar{p}=\frac{p_{1}+p_{2}}{2}=0.8$$

$$N=\frac{{\left[Z_{\alpha }\sqrt{2\bar{p}\left(1-\bar{p}\right)}+Z_{\beta }\sqrt{p_{1}\left(1-p_{1}\right)+p_{2}\left(1-p_{2}\right)}\right]}^{2}}{{\left(p_{1}-p_{2}\right)}^{2}}=47$$

The sample size would need to be 94 patients at least, and thus, 47 patients in each group.

### Randomization and grouping

A total of 100 patients were recruited and everyone signed the informed consent form. Using a predetermined randomization code via a random number generator, participants were blindly randomized into either the treatment or control group, with 50 patients in each group. The random numbers were placed in sealed envelopes, and a serial number was assigned to each envelope according to the sequence of allocation of the randomized number [17]. Each envelope was opened sequentially, according to the admission sequence of subjects at the research center. Subjects as well as physicians and nurses were blinded to the patients’ allocation. Each subject received a container marked with different colored labels (e.g., green or white) with 60 capsules (1000 mg each) inside. Detailed instructions of administrating the capsules were given to all patients. Each subject took 1 color-coded capsule orally twice a day with water. The unmasking of the container was not done until the end of the study by the Sponsor. After randomization, trained physicians were responsible for performing the clinical examinations, ordering and interoperating laboratory testing, and conducting investigations.

### Outcome measures

1) Patients’ basic information: age, sex, ethnicity, past medical history, current drug/medication use, drinking history, clinical diagnosis and the course time, family history.

2) Primary outcome measures (followed-up once before the intervention and 3 times after respectively): Five respiratory symptoms included chronic cough, chronic expectoration, asthma/dyspnea, chronic angina and chronic bronchitis or bronchitis. The respiratory symptoms scores were measured using a 5-point Likert scale which was divided (none, mild, moderate, severe and very serious) and labeled 1–5, respectively (Cronbach’s Alpha 0.71). The scores for each symptom were then added up as the respiratory symptoms scores (5–25). The higher the score, the more serious the respiratory symptoms. It can be considered as the main clinical index that results in the severity of the respiratory symptom.

3) Secondary outcome measures: Laboratory blood examinations and health survey (SF-36) evaluation (at the beginning and the end of the intervention). A standardized form was used to record the findings.

### Monitoring of patients

All subjects were asked to return to the clinic on a weekly basis for the first month and on a monthly basis for the remaining 3 months. The following tasks were performed at the time of each return visit: 1) any concerns that subjects may have were answered; 2) compliance and toxicity were evaluated; 3) capsules were resupplied.

Any serious and unexpected adverse events associated with the capsules were reviewed and examined carefully by the study physician and an unbiased written report of the event within 10 calendar days of the initial reporting was issued to the local health authority. At the minimum, the physician commented on the outcomes of the adverse event and relationships of the adverse event to the test protocol.

### Statistical analysis

Statistical data analyses were carried out using SPSS Version 11 (SPSS Inc., Chicago, IL, USA). Normality of distribution of all continuous variables was explored by a Kolmogorov–Smirnov test. A 2-group Student’s *t*-test or Mann–Whitney test was used to compare difference in means or mean ranks of variables between the control group and treatment group from baseline data. Chi-squared (*χ*^2^) test was used to compare categorical variables. For the comparison of the trend of respiratory symptoms scores between the 2 groups during 3 months of follow-up, repeated measure of analysis of variance were employed. All tests were 2-sided and a *P* value of less than 0.05 was considered to be statistically significant.

## Result

### Baseline data

One hundred subjects were recruited in this study, but 1 patient in the control group withdrew, leaving a total of 99 patients who completed the study: 50 patients in the treatment group and 49 patients in the control group. The baseline characteristics of patients regarding gender, age, history of taking medications, history of smoking, history of drinking, respiratory symptoms, physical examination, experimental detection index and SF-36 evaluation were comparable between the 2 groups (Table 1).

**Table 1:** Comparison of the baseline data between treatment and control group

**Items**	**Treatment group (n = 50)**	**Control group (n = 49)**	*P *value*
Age (years)	64.33 ± 9.41	65.90 ± 10.73	0.436
Gender (male % )	21/50 (42.0)	23/49 (46.9)	0.621
Medication history (positive %)	10/50 (20.0)	6/49 (12.2)	0.295
Smoking history (positive %)	7/50 (14.0)	9/49 (18.4)	0.555
Drinking history (positive %)	11/50 (22.0)	6/49 (12.2)	0.198
Respiratory symptoms score	8.86 ± 2.15	8.92 ± 2.33	0.897
Chronic cough (positive %)	46/50 (92.0)	47/49 (95.9)	0.692
Chronic expectoration (positive %)	43/50 (86.0)	43/49 (87.8)	0.796
Asthma/Dyspnea (positive %)	14/50 (28.0)	18/49 (36.7)	0.353
Chronic angina (positive %)	20/50 (40.0)	19/49 (38.8)	0.901
Chronic bronchitis (positive %)	31/50 (62.0)	27/49 (55.1)	0.486
Respiratory rate (times/min)	19.78 ± 2.33	20.33 ± 2.07	0.220
Heart rate (times/min)	80.10 ± 6.99	81.22 ± 6.99	0.426
HGB (mg/dL)	133.56 ± 12.43	129.65 ± 11.51	0.108
RBC (10 [9]/mm [3])	4.51 ± 0.47	4.43 ± 0.54	0.418
WBC (10 [9]/mm [3])	6.35 ± 1.34	6.69 ± 1.21	0.188
Neutrophil (10 [9]/mm [3])	4.49 ± 1.20	4.76 ± 1.21	0.261
Scores of life quality (SF-36)	109.98 ± 16.33	107.94 ± 13.09	0.596

*HGB*, Hemoglobin; *RBC*, Red blood cell; *WBC*, white blood cell.

*Unpaired Student’s *t*-test or Mann–Whitney test was used to compare differences in mean or mean ranks of variables between the control group and treatment group. Chi-square test was used for categorical data.

### The comparison between the 2 follow-up groups on the effects of the residual drug

As shown in Table 2, the comparison of residual drug between the 2 groups had no statistical difference both in 1 month and 2 months follow-up (*P* > 0.05).

**Table 2:** Comparison between 2 groups of residual drug in 1 month and 2 months follow-up

**Residual drug**	**One month follow-up**		**Two month follow-up**	
	**Treatment group**	**Control group**	**Treatment group**	**Control group**
0 capsules	25(50.0)	20(40.8)	38(76.0)	31(63.3)
1-5 capsules	17(34.0)	23(46.9)	6(12.0)	10(20.4)
6-12capsules	8(16.0)	6(12.2)	6(12.0)	8(16.3)
Total	50(100.0)	49(100.0)	50(100.0)	49(100.0)
Z / P value*	0.547/ 0.584		1.298/ 0.194	

* Mann–Whitney test was used to compare differences of mean rank between the control group and treatment group.

### The comparison between the 2 follow-up groups of respiratory symptoms before and after intervention

As shown in Table 3, after a 3-month intervention, the percentage of patients reported to have chronic cough, chronic expectoration and chronic bronchitis were significantly decreased in the treatment group when compared with baseline. The percentage of patients reported to have chronic expectoration was also decreased in the control group. The improvement of chronic cough was also significant in the treatment group versus the control group. No significant difference was found for other respiratory symptoms between the 2 groups after a 3-month intervention. No serious or unexpected events associated with the capsules were found in the intervention.

**Table 3:** Comparison of positive respiratory symptoms between treatment group and control group within a 3-month intervention

**Items**	**Treatment group (n = 50)**	**Control group (n = 49)**	***P *value**^**#**^
Chronic cough (positive %)			
Baseline	46/50 (92.0)	47/49 (95.9)	0.692
3 month after	38/50 (76.0)*	45/49 (91.8)	0.032^#^
Chronic expectoration (positive %)			
Baseline	43/50 (86.0)	43/49 (87.8)	0.796
3 month after	35/50 (70.0)*	37/50 (75.5)*	0.538
Asthma/Dyspnea (positive %)			
Baseline	14/50 (28.0)	18/49 (36.7)	0.353
3 month after	12/50 (24.0)	15/49 (30.6)	0.460
Chronic angina (positive %)			
Baseline	20/50 (40.0)	19/49 (38.8)	0.901
3 month after	21/50 (42.0)	16/49 (32.7)	0.336
Chronic bronchitis (positive %)			
Baseline	31/50 (62.0)	27/49 (55.1)	0.486
3 month after	25/50 (50.0)*	26/49 (53.1)	0.761

* *P* < 0.05 when comparing these variables before versus after intervention in the treatment group or control group by using paired sample chi-square.

^#^*P* < 0.05 when comparing these variables between treatment group and control group by using chi-square.

### Repeated measurements comparison of respiratory symptoms scores

Respiratory symptoms were assessed and scored 4 times: 1) at enrollment baseline; 2) 1-month follow-up; 3) 2-month follow-up; 4) and the end of follow-up. Repeated measure of analysis of variance was used to assess the effect of change with the lapse of time after treatment and the effects of treatment in the intervention and control groups. There were differences (*P* = 0.002) among repetitive measurement of the treatment group at 4 time points, the scores declined gradually with the lapse of time, and kept stable (*P* < 0.001) after 2 months follow-up. The follow-up score changes also had statistical significance when compared to the baseline scores. There were no significant score changes with the lapse of time in the control group (*P* = 0.082), but compared with the baseline, the first scores after 1 month of the control group were lower and remained stable after 1 month. The cause of the score decline may be due to the placebo effect. There were no significant differences between the 2 groups by test of between-subjects effects of repeated measure (*P* = 0.409) (Table 4 and Figure 1).

**Table 4:** **The comparison of respiratory symptoms scores between treatment and control group by repeated measure of analysis of variance (**$\bar{x}\pm s$**)**

**Group**	**Baseline**	**One month follow-up**	**Two month follow-up**	**End of follow-up**	**F**	P*
Treatment	8.86 ± 2.15	8.24^#^ ± 2.33	7.82^#^ ±1.88	7.92^#^ ±1.99	6.582	0.002*
Control	8.92 ± 2.33	8.36 ± 2.00	8.45 ± 2.41	8.39 ± 2.33	2.279	0.082
F P	0.689 0.409	—	—	—		—

* *P* < 0.01 when assessing the effect of change with the lapse of time by using repeated measure of analysis of variance.

# *P* < 0.05 when comparing the scores of follow-up versus of baseline by using paired sample *t*-test.

**Figure 1:**
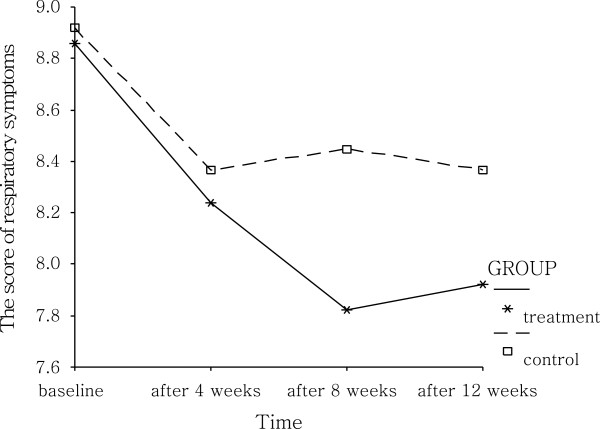
Comparison of respiratory symptoms scores between groups.

## Discussion

Chinese herbal medicine has become increasingly popular around the world for the promotion of health and adjuvant therapy [18-20]. To our knowledge, this prospective clinical research was the first study to assess the effects of Lung Support Formula, the nutritional supplement containing natural Chinese herbs, on the improvement of respiratory symptoms in older adults. The findings from this study suggested that Lung Support Formula might be beneficial to older adults by improving respiratory symptoms. The treatment was well-tolerated and none of the observed adverse events were considered drug related. There were obvious improvements in the respiratory symptoms in the treatment group; the total scores show a decreasing tendency with progress of drug taking, and the severity of symptoms presented a tendency to be gradually relieved. Based on the concrete symptoms, Lung Support Formula is useful for improving chronic cough, chronic expectoration and chronic bronchitis or bronchitis, but the efficacy of improving asthma, dyspnea and chronic angina is not obvious. Placebo effect was found in the control group; the total scores of respiratory symptoms decreased significantly after the first follow-up, but remained stable in the subsequent follow-up. This suggested that the placebo effect played a role in the subjective effect of the symptom scores and the change of scores were significant, but the placebo effect kept the symptom scores stable as treatment progressed. The scores of the treatment group improved gradually with supplement administration at the same period.

Lung Support Formula contains natural ingredients with multiple pharmacological properties, including A*stragalus membranaceus*, *Cordyceps sinensis*, *Ophiopogon japonicus*, *Panax ginseng*, *Morus alba* and *Ginkgo biloba*, plus vitamin A , vitamin C, magnesium and zinc which may provide greater clinical effectiveness with fewer side effects than compound-based drugs [21]. As the main pharmacological property of the Lung Support Formula capsules *Astragalus membranaceus* is used in Traditional Chinese Medicine and has been asserted to be a tonic that can improve lung functions, promote healing, and reduce fatigue [22]. *Astragalus membranaceus* has antibacterial and anti-inflammatory properties. In addition, a study has shown that astragalus has antiviral properties and stimulates the immune system, suggesting that it may help prevent colds and treat respiratory infections [23]. *Cordyceps sinensis* is another primary ingredient, which is a potentially ergogenic herb that has been used in Chinese folk medicine for centuries, primarily as a “lung invigorator” [24]. Studies have identified potential performance-enhancing benefits of cordyceps, including increased ventilation capacity [15] and improved glucose metabolism [25]. *Ophiopogon japonicus* is an important traditional Chinese herbal medicine and has many health benefits in treating a wide range of disorders [26]. Herbalists use ophiopogon to treat certain respiratory conditions because it is known to stimulate production of mucus, making coughs more productive. As we know, minor infections, especially infections like viral bronchitis, which cannot be treated with antibiotics, can be more easily cleared out of the respiratory tract if a patient has a productive cough. Current research also finds that saponin from ophiopogon can be explored as a novel and potential natural immunostimulant that may have several kinds of bioactivities, such as antiviral, anti-inflammatory, antiparasitic, immune-enhancing, anticancer, and antimicrobial activities [27]. *Panax ginseng* is a popular herbal remedy that has been used for thousands of years. It has been an important part of the pharmacopoeia of Traditional Chinese Medicine and is classified as an adaptogen that is thought to increase the body’s overall resistance to stress and infection [28]. A research study demonstrated that ginsenoside, an essential constituent of Chinese anti-asthmatic herbal medicine and aphrodisiacs, relaxes human bronchial smooth muscle presumably through a stimulation of nitric oxide generation by airway epithelium [29]. These 4 primary ingredients of Lung Support Formula are all beneficial to reduce respiratory symptoms.

Among the strengths of our research was the fact that it was a clinical study with a 3-month follow-up, allowing for more precise estimation of effects. However, the present study had several limitations. First, the sample size of our research was relatively small and the duration of study was not long enough; larger multicentre and longer-term treatment studies are warranted to verify the effects of Lung Support Formula. Second, one of the primary outcome measures was the respiratory symptom scores developed by a 5-point Likert scale, which was based on self-reporting by patients, who might underreport or overreport their symptoms. This bias cannot be determined from the data without objective measurements as in other studies [4,30,31], however, reliability study (Cronbach’s Alpha coefficient) had indicated good fitness results for the evaluation of respiratory symptom scores in our research.

Despite these limitations, the current study provides evidence of the improvement of respiratory symptoms among older adults by taking Lung Support Formula. Findings also indicate this natural product with multiple pharmacological properties is safe.

## Conclusions

This clinical research lends support to the hypothesis that the proprietary formula, Lung Support Formula, which contains naturally derived Chinese herbal medicines will lead to a significant improvement of respiratory symptoms and is well-tolerated in short-term use among older adults. An additional study involving more subjects and longer-term follow-up would be needed to provide convincing evidence of the improvement of respiratory symptoms in the treatment group.

## Abbreviations

COPD: Chronic obstructive pulmonary disease; HGB: Hemoglobin; RBC: Red blood cell; WBC: White blood cell.

## Competing interests

The authors declare that they have no competing interests in relation to the materials presented in this paper.

## Authors’ contributions

All the authors were involved in the development of the research design and conduction. All the authors read, commented and contributed to the submitted manuscript. YC drafted the manuscript and has been involved in the interpretation of the data. RS, JR, MS, KK and PG contributed the design of this research. RS and JR have been involved in revision of the manuscript through all stages. HS, MS and TS played a major role in the field survey. All authors read and approved the final manuscript.
